# Structural Design and Performance of Cut-Resistant Fabrics with Concave–Convex Arrays

**DOI:** 10.3390/polym16152137

**Published:** 2024-07-27

**Authors:** Fei Jiang, Ting Su, Leimei Fang, Kezheng Zhao, Honglian Cong

**Affiliations:** Engineering Research Center of Knitting Technology, Ministry of Education, Jiangnan University, Wuxi 214122, China; 6223017011@stu.jiangnan.edu.cn (F.J.); 6223016016@stu.jiangnan.edu.cn (T.S.); 6233017007@stu.jiangnan.edu.cn (L.F.); zhaokezheng1@outlook.com (K.Z.)

**Keywords:** concave–convex arrays structure, flat knitting, cut resistance, UHMWPE, abrasion resistance

## Abstract

As the risk of social security increases, it is crucial to develop flexible protective materials that combine flexibility with high protective performance. Ultra-high-molecular-weight polyethylene (UHMWPE) was selected as the raw material, and four types of flat-knitting cut-resistant fabrics were ultimately designed and prepared from a three-dimensional longitudinal dimension and concave–convex array structure based on rib knitting. A series of experiments must be conducted on fabrics in order to study the law of protection performance of different structural fabrics. They were thus subjected to comprehensive evaluation and theoretical analysis of cut resistance. The results demonstrate that the four structural fabrics exhibited resilience in abrasion tests, withstanding over 100,000 cycles without failure. A weighting algorithm was employed to determine the comprehensive cutting resistance of the S1, S2, S3, and S4 structural fabrics, resulting in values of 1939.9 gf, 2298.6 gf, 2577.1 gf, and 2822.2 gf, respectively. Therefore, S1 reached class A4, which is sufficient to address a medium cut hazard. Similarly, S2, S3, and S4 reached class A5, which is adequate to address a high cut hazard. The obtained fitting equation, with uniform yarn fineness T as the dependent variable, demonstrates that the cut resistance improved as the concave–convex density level increased.

## 1. Introduction

The increasing prevalence of safety risks at work and in life has led to an increased emphasis on physical safety and protection. In nature, many animals have evolved shells or armor to protect their soft bodies [1]. Inspired by this, various protective equipment, including cut-resistant gloves, bullet-proof vests, stab-proof suits, and protective helmets [2,3,4,5], have been developed to ensure that the human body can effectively mitigate or even resist stabbing and cutting injuries when impacted by sharp objects [6]. Knives, in particular, are one of the most commonly used weapons in violent incidents in society. It is notable that the majority (63.3%) of stabbing injuries are of the cut type [7]. Consequently, research on protective materials, particularly in-depth exploration of their cut resistance, is of particular importance.

A plethora of studies have been conducted on protective clothing fabrics. Four primary categories of materials have been identified as exhibiting excellent cut resistance: rigid materials comprising metal or ceramics, coated materials comprising epoxy resin compounded with woven fabrics, semi-rigid materials comprising rigid material divided into small pieces fixed on a soft substrate, and flexible fabric materials comprising high-performance fibers [8,9,10,11]. Among these materials, fibrous materials with high strength and high modulus, such as metal fibers and high-performance fibers, have become the focus of research. These include stainless steel fibers, Kevlar fibers, basalt fibers, and glass fibers [12,13]. In terms of fabric structure, protective materials are mainly interwoven by yarns, resulting in a dense structure. The woven structure is compact, with the yarns exhibiting low slippage when subjected to the action of the knife edge. However, the texture is hard. Non-woven structure fabrics are typically multilayer composites, comprising several layers of materials that are bonded together [14,15,16]. Composite structure fabrics are created through the use of coating or laminating technology, whereby multi-layer fabrics are glued together to create a single entity that can perform a variety of functions associated with composite materials [17,18]. The aforementioned fabric structures exhibit excellent protective properties yet often encounter challenges in terms of weight and softness when utilized in long-term-wear garments [19]. In the context of work and living environments, there is a growing emphasis on the comfort and lightness of garments. Consequently, the development of fabrics that combine excellent protective properties with good comfort and light weight has emerged as a current research focus [20].

The advancement of production technology and the increased output of UHMWPE yarn have led to the emergence of a new generation of high-performance special yarn, ranking alongside carbon fiber and aramid fiber. Its monofilament strength is the greatest among the current high-performance fibers, with a notable energy absorption capacity. Consequently, it exhibits a range of exceptional properties, including light weight, high strength, impact resistance, and cut resistance [21]. In comparison to the high cost of Kevlar fibers and the discomfort caused by glass fiber, protective products based on UHMWPE exhibit significant advantages. In particular, knitted fabrics woven with this fiber exhibit a unique softness of the loop, improving the stiffness of the material and greatly satisfying people’s expectations for flexible personal protective clothing [22,23]. Wang et al. conducted an investigation into the impact of UHMWPE weft-knitted fabric structure, orientation, and stacking on puncture resistance. Their findings indicated that the double-sided structure of weft-knitted fabrics and the course direction of the fabrics exhibited enhanced puncture resistance [24]. Tan et al. developed two kinds of reinforcing yarns for three-dimensional planar-knitted fabrics using UHMWPE yarns. The results demonstrated that the fabrics with interlocked structures exhibited superior performance compared to those with planar structures [25].

Although knitted fabrics are flexible and lightweight, their cut resistance is not as good as that of woven fabrics, limiting their application in areas with high protection needs. It is therefore evident that there is a need to develop methods of improving cut resistance while maintaining the softness and comfort of knitted fabrics. The objective of this paper is to enhance the cut resistance of knitted fabrics by optimizing their organizational structure while maintaining their characteristic flexibility and light weight. Given the structural flexibility and design convenience of flat-knitted fabrics [26], this paper focuses on them with the aim of realizing the enhancement of cut resistance. Accordingly, four process structures were designed using UHMWPE as a raw material and based on the double-sided rib combination stitch. The fabrics formed an obvious concave–convex array structure, resulting in four cut-resistant fabrics. A comprehensive analysis of the impact of the fabric concave–convex array structure on the fabric protection performance was conducted to ascertain the influence of the law. These fabrics are suitable for public safety applications such as plainclothes police and security personnel as well as industrial scenarios such as machine operation and metal processing. They provide effective protection against knife cuts or mechanical injuries while maintaining the flexibility and comfort of the wearer [27,28]. This paper presents new ideas and methodologies for the development of protective materials for knitted fabrics, with the objective of advancing the creation and implementation of high-performance protective materials.

## 2. Materials and Methods

### 2.1. Design Principles

**Figure 1:**
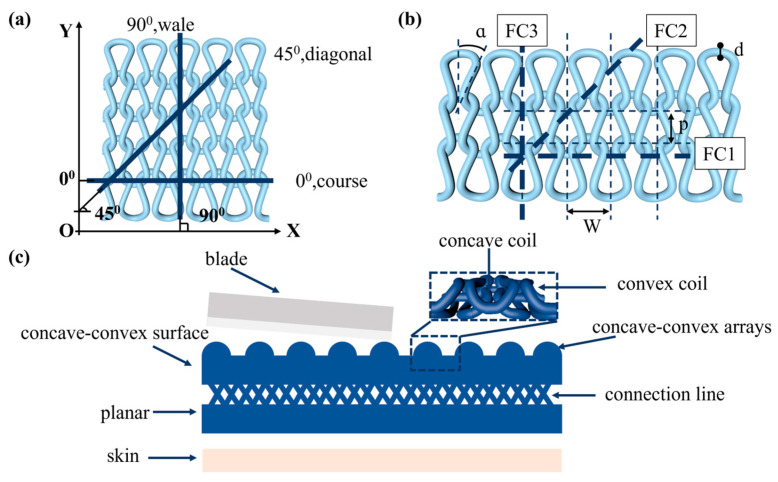
Design basis for cut-resistant fabrics. (**a**) Schematic diagram of the cutting angle of fabric resistance; (**b**) schematic diagram of the fabric cut resistance equation; (**c**) fabric design principle.

The cutting of fabrics is performed with the use of knives, resulting in a line contact between the knives and the fabric surface. The cutting length is defined as the blade slip distance, while the cutting depth is measured along the thickness direction of the fabric [29]. The cutting angle of the tool and the knitted fabric can also influence the cut resistance of the fabric. The cutting angle is primarily oriented along the fabric’s course, diagonal, and wale directions, as illustrated in Figure 1a. Messiry developed equations to predict the cut resistance of weft-knitted fabric units based on the directions of the cutting angle [30]. The cutting resistance of the fabric can be calculated by multiplying the cutting resistance equations for different cutting directions by the number of fabric loops at the cutting length of the tool in the corresponding cutting direction (Figure 1b).

(1) $$FC1=\pi d^{2}\tau \left(0\right)$$

(2) $$FC2=\pi d^{2}\tau \left(0\right)+Ld\tau \left(90\right)$$

(3) $$FC3=0.5\pi d^{2}\tau \left(0\right)$$

(4) $$L={\left(p^{2}+d^{2}\right)}^{0.5}$$

(5) $$\tan\alpha =\left[\left(0.5W+d\right)/2p\right]$$

where *d* is the yarn diameter, *τ* (0) is the shear stress when the tool is perpendicular to the yarn cross-section, *τ* (90) is the shear force when the tool cuts along the yarn axis, *L* is the loop column, *p* is the loop cross-column spacing, *W* is the longitudinal spacing of the loops, *α* is the inclination of the loop column with respect to the *y*-axis, and FC1, FC2, FC3 are the cutting forces along the 0°, 45°, and 90° directions.

When the tool and experimental conditions are consistent, and the fabric yarn strength is sufficiently strong, the key to improving the cutting resistance of the fabric is to increase the number of fabric loops under the cutting length of the tool and the contact area between the yarn and the tool per unit length [31]. UHMWPE filaments were selected as the main body to ensure that the cut resistance of the fabric was strong enough, with nylon/spandex core yarns employed to facilitate the shrinkage of the UHMWPE fabric. In the plane dimension of the fabric, the transverse and longitudinal densities are typically tightened to increase the number of fabric loops, which in turn increases the contact area between the tool and the fabric. In this paper, the organization structure of the fabric in the three-dimensional longitudinal dimension is presented as a concave–convex array structure. When cut by the tool, the loops intertwine with each other and stack, thereby increasing the area of contact between the fabric and the tool. This, in turn, achieves an exponential increase in the cut resistance of the fabric unit area. In order to reduce the influence of fabric thickness and gram weight on cut resistance, the fabric was designed to be concave–convex on one side and planar on the other side. The main research objective is to study the influence of the concave–convex side, i.e., the front side of the fabric process, on the fabric’s protective performance. The fabric design principle is shown in Figure 1c.

### 2.2. Fabric Structure Design

In the course, loop-forming knitted fabric structure, the rib stitch, with its double-sided stitch, exhibits greater puncture resistance. In contrast, the interlock stitch is composed of a two-rib stitch, resulting in a fabric with greater density and thickness than that of the rib stitch [32,33], which is more suitable for the production of stiff outerwear fabrics.

**Figure 2:**
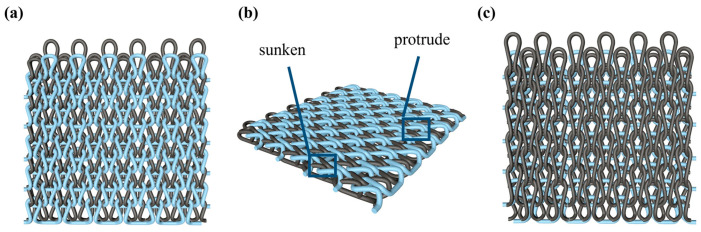
Fabric concave–convex arrays structure diagram. (**a**) Front of fabric; (**b**) concave–convex arrays structure; (**c**) back of fabric.

As illustrated in Figure 2, this double-sided stitch comprises a rib stitch that is employed to establish a robust connection between the front and back sides. In contrast, the variegated flat-needle stitch is knitted exclusively on the front side (Figure 2a,c). The introduction of the variegated flat-needle stitch results in an increase in the number of knitting rows and a concave joining point for the rib stitch due to the cross-row pulling of loops on the reverse side. This creates a convex front side. The side loops are pulled tightly together, and the fabric at the variegated flat-needle stitch is relatively convex, without any connection between the fabrics (Figure 2b), which reflects the concave–convex array effect [34]. By varying the knitting row order and the number of rows of flat-knit stitch, the degree of structural concavity and convexity can be achieved, resulting in an orderly and quantifiable gradient design.

**Figure 3:**
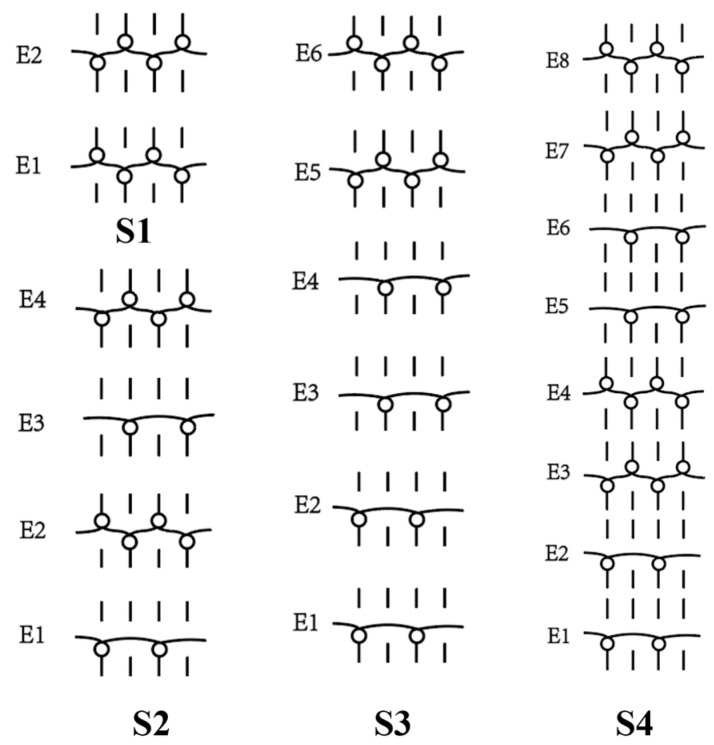
Fabric knitting process.

In this paper, the concave–convex arrays are refined into concave–convex densities, which indicate the overall effect of the degree of concave–convex densities of the structures under the arrays. Four process organization structures were designed, and the fabric knitting process is illustrated in Figure 3. These structures are designated as S1, S2, S3, and S4.

This paper introduces a variation of the flat stitch, namely the one-over-one flat-needle stitch. Structure S1 is an interlock stitch composed of the rib stitch. The two sides of the structure are closely connected, and the loops are concave–convex arrays. Structure S2 is based on structure S1 and introduces the one-over-one flat-needle stitch. This is knitted only on the front side and is knitted in interlocked rows with the rib stitch. The concave–convex arrays structure is staggered. The fabric exhibits a small twill array. Structure S3 builds upon structure S2 by increasing the number of knitting rows of one-over-one flat-needle stitch, intensifying the density of concave and convex fabrics and showcasing a small checkered concave–convex array. Structure S4 is based on structure S3 and involves an adjustment in the order of the knitting rows of the rib stitch and one-over-one flat-needle stitch, resulting in the most obvious intensification of the concave–convex density of the fabric, which presents large checkered concave–convex arrays.

As shown in Table 1, the loops of each organization structure are tightly connected to each other, as seen from the coil simulations on the front and back sides of the fabric. When studying the concave–convex array structure characteristics of the stitch, in order to simplify the analysis, we implemented an innovative simplification strategy. All concave–convex array structures in this paper are considered as a unified staggered concave–convex array arrangement pattern. This treatment not only streamlines the analytical process but also establishes a foundation for subsequent quantitative work. On this basis, two basic unit structures are defined: the unit concave structure and unit convex structure. To quantify the degree of aggregation of these concave–convex structures, a numerical expression was used, and the value of 1 was taken as the planar reference point. Based on the comprehensive consideration of the size of the loops and the number of loops within the unit structure, comparing the differences between the different structures and the reference point, the concave–convex density distribution maps on the face of all the stitches were obtained. The distribution map serves to illustrate the concave–convex characteristics of the fabric surface while simultaneously providing robust support for subsequent calculations and analysis. In order to more precisely define and classify the levels of different fabrics in terms of concave–convex density, we calculated uniform values as quantitative indexes based on the concave–convex density distribution map. For example, the unit convex structure of S2 is larger than the unit planar structure of S1, while the unit concave structure of S2 is only slightly smaller than the unit planar structure of S1. The set values are integers; therefore, the unit concave structure of S2 is considered to be 1 and the excess gap assigned to the unit convex structure considered to be 2. Similarly, S3 treats the unit concave structure as 1, and the excess gap is assigned to the unit convex structure, which is considered as 3 due to the presence of two loops within the unit convex structure. Similarly, the concave–convex densities distribution map for S4 was identified. Subsequently, the concave–convex density level of the fabric can be obtained by calculating the numerical average of the four grids in the concave–convex density distribution map, which represents a staggered concave–convex array arrangement pattern. Therefore, structure S1 is flat, and the density level is defined as R1 level; structure S2 has a slightly higher density and is defined as R1.5 level; structure S3 has an increased density and is defined as R2 level; and structure S4 has the most significant density and is defined as R3 level. The main investigation studies the effect of different structures of fabric frontal bump density levels on fabric properties.

### 2.3. Fabric Preparation and Test Method

The fabrics were knitted on a domestic Lonestar KSC-132 computerized flat-knitting machine (Jiangsu Jinlong Science and Technology Co., Ltd., Suzhou, China), with the on-machine program pre-drawn on the accompanying KDS-MAIN software. A nylon/spandex (70/30) core yarn (yarn fineness 111.1 dtex, 77.8 dtex nylon outer, 33.3 dtex spandex core; Engineering Research Center of Ministry of Education for Knitting Technology, Jiangnan University, Wuxi, China) was threaded into the yarn feeder and knitted synchronously with a UHMWPE filament (yarn fineness 666. 7 dtex/240 f, no twist; Kyushu Star Technology Co., Ltd., Nantong, China) in order to synchronize the knitting and avoid the loosening of the fabric knitted with the essentially inelastic UHMWPE. Through the sample, the on-machine density of the fabric was slightly adjusted so that the thickness and grammage difference of the fabric after the off-machine was small. The main study was of the protective performance of the concave and convex surface of the fabric, so the experiments on the fabric abrasion resistance and cut resistance were carried out from the front side of the fabric process.

The abrasion resistance of fabrics is defined as the ability of fabrics to resist abrasion. Abrasion is a process whereby fabrics are gradually damaged by repeated friction from other objects, often during the process of use [35,36]. In this paper, the abrasion resistance of fabrics was mainly measured by its flat abrasion number, which represents the extent to which the appearance or performance of fabrics is maintained after being subjected to a certain number of abrasions. The abrasion resistance test was based on GB/T21196.2-2007 “Textiles—Determination of the abrasion resistance of fabrics by the Martindale method—Part: Determination of specimen breakdown” [37], and the instrument used a YG401G type fabric flat abrasion tester, i.e., a Martindale tester (Ningbo Textile Instrument Factory, Ningbo, China), to test the abrasion resistance of fabrics under 9 Kpa. At the same time, in order to compare the abrasion resistance of four kinds of structural fabrics more intuitively, the YG522N fabric abrasion resistance tester (Shanghai Huyeming Scientific Instrument Co., Ltd., Shanghai, China) was used to conduct the abrasion resistance experiments in the case of pressurized 1500 g weights (750 g weights + 750 g weights) and a pair of abrasive wheels as abrasive material. The fabric was abraded 3000 times, and the abrasion resistance index was calculated according to Equation (6).

(6) $$Ai=\frac{n}{∆m}$$

where *Ai* is the abrasion resistance index, times/mg; *n* is the total number of abrasion times, times; Δ*m* is the mass loss of the specimen under the total number of abrasion times, mg.

**Figure 4:**
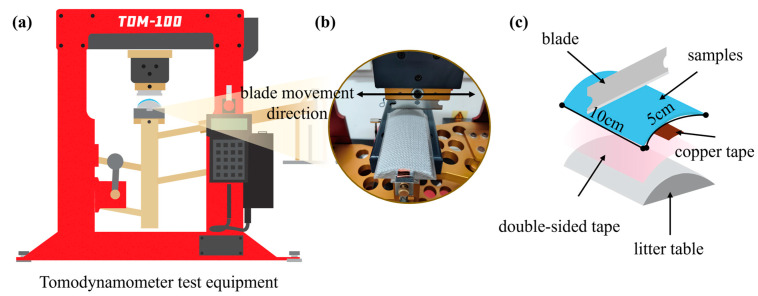
Fabric cut resistance test. (**a**) Tomodynamometer test equipment; (**b**) schematic diagram of blade cutting on fabric and cutting direction; (**c**) preparation method for cutting samples.

A cut resistance test was conducted in accordance with the F2992/F2992M-15 “Standard Test Method for Measuring Cut Resistance of Materials Used in Protective Clothing with Tomodynamometer (TDM-100) Test Equipment” [38] and ANSI/ISEA 105-2016 “American National Standard for Hand Protection Classification” standards [39], using a TDM cut resistance tester (Figure 4a,b) (Shanghai ANYI Scientific Instrument Co., Ltd., Shanghai, China). When the fabric is cut by the blade, the electrical signal transmitted by the contact of the blade with the copper tape stops the blade immediately, and the sensor indicates the distance of the blade movement (Figure 4c). Four distinct fabrics were subjected to cutting tests. The cutting test was carried out at three angles (0°, 45°, and 90°) with a tool slip distance of 5–20 mm, 20–33 mm, and 33–50.8 mm. Five points were taken at each interval, and the data were subjected to inverse linear regression analysis. The fabric was deemed to have been cut when the tool slip distance was 20 mm, and the resulting cutting force was used as a rating standard.

## 3. Results and Discussion

### 3.1. Fabric Characterization

The fundamental characteristics of the fabrics following shrinkage and sizing are presented in Table 2. The thickness of the four structural fabrics is less than that of the protective fabrics currently available on the market (UHMWPE high-density woven fabric is 2 mm), and the overall discrepancy in thickness is not significant, with a range of 1.60~1.75 mm. In the gradient, the difference in surface mass is 10 to 15 g/m^2^, which is approximately consistent with the surface mass of 645 to 675 g/m^2^. The four structural fabrics exhibit similar course density, which is 36 wale/(5 cm). Due to differences in organizational structure and adjustments for similarity in surface mass, the wale density varies among the fabrics.

**Figure 5:**
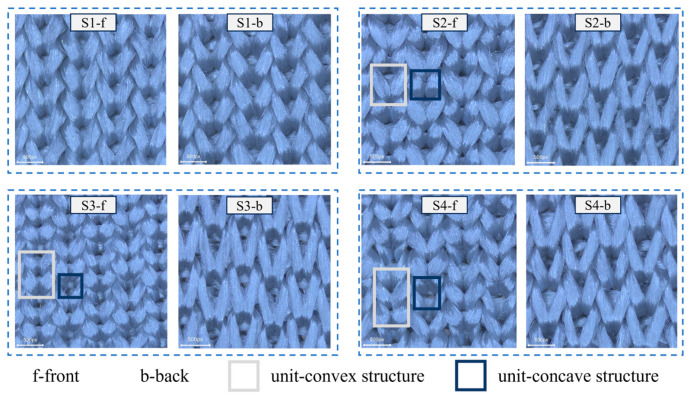
Fabric object picture.

The four flat-knitting concave–convex array cut-resistant fabrics are shown in Figure 5 under the body-view microscope, with the front side of the fabric on the left and the back side of the fabric on the right. The light box indicates the unit convex structure under the fabric concave–convex arrays, and the dark box indicates the unit concave structure under the fabric concave–convex arrays. Due to the shrinkage of the fabric after the machine, there is still a gap with the effect of the loop simulation. The actual effect of the four structures of fabrics S1, S2, S3, and S4 is in line with expectations. Upon examination of the front side of the fabric process, it was evident that the size of the loops is influenced by the interaction of the rib stitch and the one-over-one flat-needle stitch. Additionally, the concave–convex densities of the fabric exhibit a specific order of increase. In contrast, the reverse side of the process is characterized by uniform size of the loops and varied flat-needle stitch.

### 3.2. Performance of Abrasion Resistance

**Figure 6:**
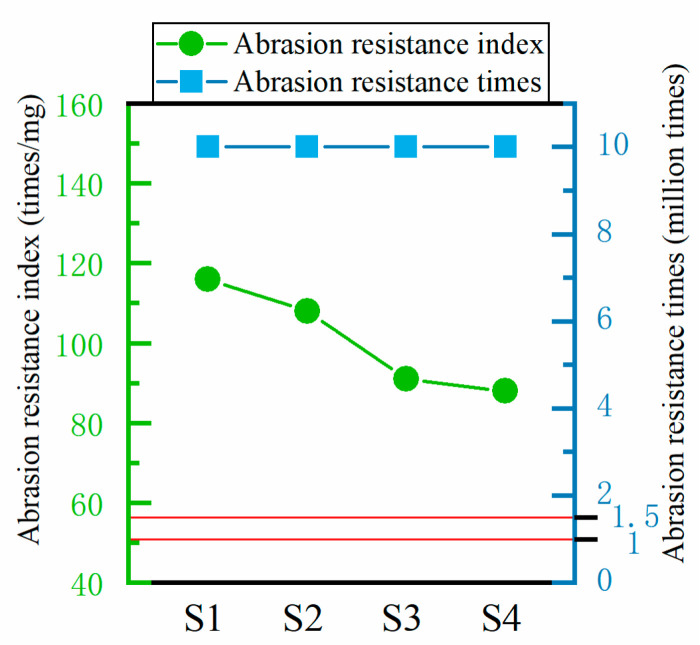
Fabric abrasion resistance.

As illustrated in Figure 6, following the 100,000-cycle Martindale meter experiment, the four structural fabrics exhibited only slight hairiness and a dull luster. In accordance with the GB/T 21295-2014 standard, “Requirements of physical and chemical performance of garments”, ref. [40], it is specified that the upper fabric must withstand at least 10,000 abrasions, while the lower fabric must withstand at least 15,000. The experiments presented in this paper demonstrate that the abrasion resistance of the fabric is in excess of 100,000 times, indicating that the abrasion resistance of the concave–convex array flat knitting fabric is exceptionally robust, exceeding the requirements set forth in the clothing standard.

The four types of structural fabrics met the abrasion resistance standard, and under the premise of excellent abrasion resistance, the influence law of abrasion resistance of fabrics was further explored. In accordance with the conditions of the Taber-type abrasion test, which represents a more severe abrasion resistance environment, the mass loss of the fabric was quantified. After 3000 cycles of abrasion, the mass loss of the four fabrics, designated as S1, S2, S3, and S4, was observed to be 25.8 mg, 27.7 mg, 32.9 mg, and 34.1 mg, respectively. The percentage of weight loss in relation to the original weight was observed to be 0.31%, 0.32%, 0.35%, and 0.41%, respectively. These findings suggest that after 3000 abrasion cycles, the fabric exhibits minimal deterioration in quality and demonstrates excellent abrasion resistance. The abrasion resistance indices of the four structural fabrics were calculated by entering the data into the Equation (6). As illustrated in Figure 6, the abrasion resistance index of structure S1 is the highest, reaching 116 times/mg. The abrasion resistance indices of S2, S3, and S4 are reduced compared with A, with values of 108 times/mg, 91 times/mg, and 88 times/mg, respectively. The gap between the three is relatively small, with a moderate decreasing tendency. Overall, the abrasion resistance index of the fabric decreases as the concave–convex density increases. It can be postulated that the concave–convex arrays structure of fabrics will increase the actual friction area on the fabric surface, resulting in an uneven distribution of friction on the fabric surface. This will lead to greater pressure being exerted on the convex areas, which will increase the abrasion in these areas. At the same time, the concave–convex arrays structure of the fabric will change the wear mechanism from smooth wear to abrasive or cutting wear, which will lead to faster wear. In contrast, structured fabrics are characterized by flat surfaces and a greater degree of durability. In general, the concave–convex array structure of the fabric will have a detrimental impact on the abrasion resistance in a high-friction environment. Therefore, it is crucial to regulate the optimal density of concave and convex elements in the subsequent optimization of the fabric structure to minimize the adverse effect on the wear resistance performance.

### 3.3. Performance of Cut Resistance

The cut resistance of fabrics is primarily determined by the cut resistance of the yarns in the fabric, organizational parameters, and experimental conditions. Among these factors, the cutting direction has a significant impact on the cut resistance of fabrics. Knitted fabrics exhibit anisotropy, with distinct yarn interweaving patterns and varying strength distributions in different directions [41,42]. The cut resistance under the 0° direction of the blade along the fabric is defined as the 0° cut resistance, and the other two directions are considered to be analogous. In different directions, there are different angular relationships between the cutting line of the blade and the yarn of the fabric loop, which is manifested as different cutting contact areas between the blade and the yarn.

#### 3.3.1. 0° Cut Resistance

**Figure 7:**
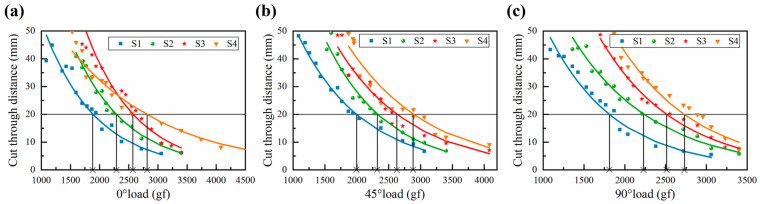
Fabric cut resistance regression line. (**a**) Fabric 0° cut resistance regression line; (**b**) fabric 45° cut resistance regression line; (**c**) fabric 90° cut resistance regression line.

The 0° cutting load is defined as the resistance encountered by the blade or cutting tool when cutting in the course direction of the knitted fabric (i.e., the direction parallel to the course direction of the fabric). When cutting along the 0° direction, the blade is positioned in a more orthogonally perpendicular manner relative to the fabric loop yarns. As illustrated in Figure 7a, the 0° cutting resistance of S1, S2, S3, and S4 is 1898.4 gf, 2287.2 gf, 2554.8 gf, and 2799.5 gf, respectively. The overall trend is a gradual increase, and the relative increase in cutting resistance for S2, S3, and S4 in comparison to that of S1 is 20.5%, 34.6%, and 47.5%, respectively. It is noteworthy that the four structural fabrics exhibit similar transverse densities, indicating that the number of loops in the course direction remains consistent. The observed discrepancy in cutting resistance can be attributed to the concave–convex array structure, which enhances the interweaving of loops in the course direction, filling up numerous holes and gaps in the knitting structure. The concave–convex array structure also increases the cutting area that the tool touches. When the fabric is flat, the cutting area is flat. However, when the fabric has a concave–convex array structure, the cutting area is a three-dimensional curved surface.

#### 3.3.2. 45° Cut Resistance

In the context of a realistic cutting hazard, the cutting action of the blade on the fabric exhibits a greater variety of oblique cutting angles. This paper selected the typical 45° direction for the evaluation of cutting resistance. When cutting in the 45° direction, there is a specific angular relationship between the cutting line of the blade and the yarn of the fabric loop, which leads to an increase in the cutting contact area and significantly enhances the cutting resistance of the fabric. As illustrated in Figure 7b, the 45° cutting resistance of S1, S2, S3, and S4 is 2010.5 gf, 2328.2 gf, 2611.4 gf, and 2862.9 gf, respectively. With the increase in the concave–convex density level, the 45° cutting resistance is also increased to some extent. In the 45° orientation of the loop, the physical properties combine the advantages of knitted fabrics at 0° and 90°, with a tighter and more uniform interweaving of the loop, providing a more concentrated force to cope with knife cuts.

#### 3.3.3. 90° Cut Resistance

The knife cuts along the fabric in a 90° direction, and since the thickness of the blade is smaller than the yarn fineness, there is a high probability that the blade will slip with the yarn and land on the needle’s knitting arc or the sinker arc or between the two loop columns. As shown in Figure 7c, the experimental data were analyzed by inverse linear regression to obtain the regression curve, and the 90° cutting resistance of the fabric was calculated. The 90° cutting resistance of fabrics S1, S2, S3, and S4 was found to be 1825.8 gf, 2241.9 gf, 2524.7 gf, and 2754.5 gf, respectively. The overall trend is one of gradual increase, with the increment from S1 to S2 being particularly notable. This is due to the fact that fabric S1 is an interlock stitch, which has an obvious longitudinal bar effect in the wale direction of the fabric. In the longitudinal direction, the loops are arranged consistently with less interweaving, which leads to the cutting load being concentrated in the longitudinal direction, increasing the risk of the yarn being cut off at one time in the longitudinal direction. The designed S2, S3, and S4 structure with interleaved small and large loops serves to fill the gap in the wale direction, thereby forming a twill interleaved array arrangement with even interweaving of yarns between the loops.

#### 3.3.4. Comprehensive Evaluation of Cut Resistance

**Figure 8:**
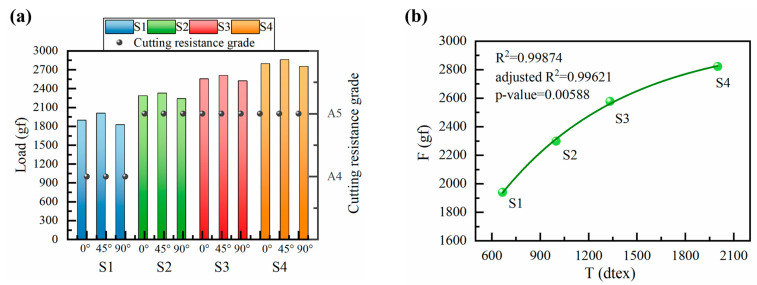
Comprehensive fabric cut resistance. (**a**) Comprehensive evaluation of cut resistance of fabrics; (**b**) fabric cut resistance correlation fitting curve.

As illustrated in Figure 8a, a preliminary assessment of the cutting resistance of each structural fabric along the three directions of 0°, 45°, and 90° was made based on the protection level recognized by the ANSI/ISEA 105-2016 standard [39]. This comparison allowed for a rough estimation of the level of cut resistance of the four fabrics. With regard to the fabric itself, a consistent trend in cutting angles was observed, with minimal variation in cutting resistance across the three angles. This is in contrast to the cutting resistance observed along the 45° direction, which is significantly higher than that observed along the 0° direction and 90° direction. This is due to the differing contact areas when the blade cuts along different directions. It was observed that the gap in cutting resistance along the three directions of the S1 structure fabric is larger than that of the other three structure fabrics. This is due to the longitudinal stripe effect of the interlock structure and the design of the concave–convex arrays structure, which makes the fabric structure interwoven tightly, with the loops staggered and uniformly aligned in the three directions.

To obtain a more accurate evaluation of its performance, a weighting algorithm was employed to synthesize the cutting resistance of the fabric in all directions, thus providing a comprehensive cutting resistance rating result [35]. The specific values of the weights were determined based on the measured data of the cutting load in different directions and were closely aligned with the requirements of practical application scenarios. Consequently, the weighting factors for 0°, 45°, and 90° cutting resistance are 0.3, 0.5, and 0.2, respectively. The integrated cutting resistance of S1, S2, S3, and S4 structural fabrics is 1939.9 gf, 2298.6 gf, 2577.1 gf, and 2822.2 gf. In summary, the cutting resistance is ranked as S4 > S3 > S2 > S1. Subsequently, S1 reached the A4 level, which is indicative of a medium cutting hazard, while S2, S3, and S4 reached the A5 level, which is indicative of a high cutting hazard. From the perspective of the concave–convex density on the front side of the fabric, it can be observed that S2, S3, S4 > S1, which indicates that the concave–convex density has a positive effect on the cut resistance of the fabric.

### 3.4. Theoretical Analysis of Cut Resistance

Concave–convex density represents a crucial element in the evaluation of cut resistance in fabrics. In order to gain a more precise understanding of this phenomenon, it is necessary to quantify the concave–convex density. The central role of a blade in the cutting of fabric is reflected in the cutting of fabric yarns. Therefore, in order to evaluate the cutting effect in a more intuitive manner, we translated the concave–convex density level into the metric of uniform yarn fineness of the fabric. The uniform yarn fineness is employed as an intermediate quantity for the purpose of establishing a correlation between concave–convex density and fabric cut resistance. In the process of fabric structure design, the frontal concave–convex density of the four structured fabrics was meticulously graded in order to more accurately reflect their overall yarn fineness. It is known that the yarn fineness T of structure S1 is 666.7 dtex; i.e., R1 is quantized as T_S1_ = 666.7 dtex, and then, the concave–convex density values of structures S2, S3, and S4 are quantized by R1.5 as T_S2_ = 1000.1 dtex, by R2 as T_S3_ = 1333.3 dtex, and by R3 as T_S4_ = 2000.1 dtex. The quantized values of the concave–convex density T of the four structured fabrics were plotted against the combined cut resistance F of the fabric, and nonlinear curve fitting was performed. The fitted curve of the correlation between the fabric concave–convex density and cut resistance is shown in Figure 8b. As can be seen from the figure, the difference between the fitted curve and the original data is very small. In addition, the R2 and adjusted R2 of the present model are values very close to 1, indicating that the model explains about 99.62% of the variation in the data, which is a high value, and the model is considered to be well fitted. Also, the *F*-test corresponds to a probability value (*p*-value) much smaller than the commonly used significance level (e.g., 0.05) [43], which strongly suggests that there is a significant relationship between the explanatory variables and the dependent variable in the model; i.e., the model is meaningful. Therefore, when the quantitative value of concave–convex density, namely the uniform yarn fineness *T*, is used as the dependent variable, the equation can be given as follows (Equation (7)):

(7) $$F=-2561.36\times e^{\frac{-T}{777.64}}+3022.30$$

It was observed that as the uniform yarn fineness *T* increased, the cut resistance *F* also increased, but the rate of increase (i.e., the slope) decreased. This phenomenon can be attributed to the fact that the increase in yarn fineness leads to an increase in the number of fibers per unit length, which in turn increases the inter-fiber interactions and holding forces and significantly improves the overall strength of the yarn. However, as the number of fibers continues to increase, the increase in inter-fiber holding force gradually slows down, and the monofilaments of UHMWPE yarns are easily disentangled. Therefore, further increase in uniform yarn fineness no longer has a significant effect on the improvement of cut resistance. It is therefore necessary to conduct further research on the variable of yarn fineness in order to ascertain whether a change in yarn fineness will have a significant impact on the cut resistance of fabrics due to its effect on the physical properties of yarns and the overall structure of fabrics. Furthermore, it is of great importance to investigate the optimal level of yarn fineness for the cut resistance of fabrics in all aspects in order to provide a solid experimental foundation for the study of flexible protective knitted fabrics.

## 4. Conclusions

In order to study and compare the laws of protection performance of different structural fabrics, this study designed and developed four kinds of structural fabrics and carried out comprehensive evaluation and theoretical analysis of the cut resistance of each fabric.

The surface of the four structured fabrics subjected to the Martindale method of abrasion resistance testing after being subjected to over 100,000 abrasions exhibited only linting and a reduction in gloss, while the fabrics remained intact, exceeding the standard for garment abrasion resistance. The mass loss measurements, conducted in accordance with the established abrasion resistance standard, revealed a correlation between the concave–convex density and the abrasion resistance index of the fabric. Specifically, it was observed that as the density of the concave–convex density increased, the fabric exhibited a decline in its abrasion resistance. This suggests that the concave–convex array structure of the fabric may have a detrimental impact on its overall abrasion resistance.

The combined cutting resistance of the S1, S2, S3, and S4 fabrics, as determined by the weighting algorithm, is 1939.9 gf, 2298.6 gf, 2577.1 gf, and 2822.2 gf, respectively. Subsequently, S1 attained the level of A4, which is defined as a medium cutting hazard, while S2, S3, and S4 reached the level of A5, which is defined as a high cutting hazard. Consequently, the bump density level exerted a positive influence on the fabric cut resistance. By quantifying the bump density level as the uniform yarn fineness *T* of the fabric and correlating it with the fabric’s integrated cut resistance *F*, a nonlinear fitting curve with a high degree of fit and a fitting equation when the uniform yarn fineness *T* was used as the dependent variable were obtained. The results demonstrate that an increase in the concave–convex density level is associated with an improvement in cut resistance. However, the rate of improvement, i.e., the slope, was observed to decline gradually.

## Figures and Tables

**Table 1:** Fabric concave–convex arrays structure distribution.

Fabric No.	Fabric Front	Fabric Back	Fabric Front Bump Density Distribution
S1	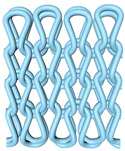	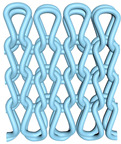	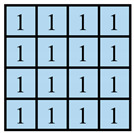
S2	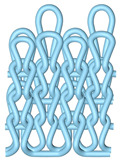	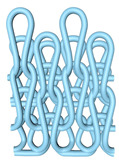	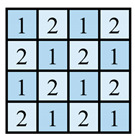
S3	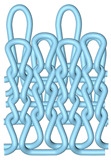	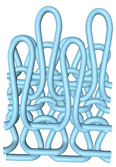	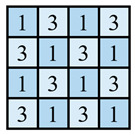
S4	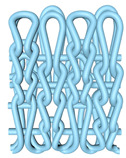	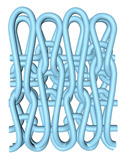	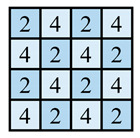

**Table 2:** Fabric basic parameters.

Fabric No.	Thickness /mm	Surface Mass/ (g/m^2^)	Course Density/ (Wale/5 cm)	Wale Density/ (Course/5 cm)
S1	1.61	645.65	36	56
S2	1.64	652.12	36	66
S3	1.69	668.47	36	76
S4	1.73	673.67	36	72

## Data Availability

Data are contained within the article.
